# Distributions of Aβ42 and Aβ42/40 in the Cerebrospinal Fluid in View of the Probability Theory

**DOI:** 10.3390/diagnostics11122372

**Published:** 2021-12-16

**Authors:** Piotr Lewczuk, Jens Wiltfang, Johannes Kornhuber, Anneleen Verhasselt

**Affiliations:** 1 Department of Psychiatry and Psychotherapy, Universitätsklinikum Erlangen and Friedrich-Alexander Universität Erlangen-Nürnberg, 91054 Erlangen, Germany; Johannes.Kornhuber@uk-erlangen.de; 2 Department of Neurodegeneration Diagnostics, Medical University of Białystok and Department of Biochemical Diagnostics, University Hospital of Białystok, 15-269 Białystok, Poland; 3 Department of Psychiatry and Psychotherapy, University Medical Center of Göttingen (UMG), 37075 Göttingen, Germany; Jens.Wiltfang@med.uni-goettingen.de; 4 German Center for Neurodegenerative Diseases (DZNE), 37075 Göttingen, Germany; 5 Neurosciences and Signaling Group, Department of Medical Sciences, Institute of Biomedicine (iBiMED), University of Aveiro, 3810-193 Aveiro, Portugal; 6 Center for Statistics, Data Science Institute, Hasselt University, 3590 Hasselt, Belgium; Anneleen.Verhasselt@uhasselt.be

**Keywords:** amyloid β, Alzheimer’s disease, probability theory, distribution of a random variable

## Abstract

Amyloid β 42/40 concentration quotient has been empirically shown to improve accuracy of the neurochemical diagnostics of Alzheimer’s Disease (AD) compared to the Aβ42 concentration alone, but this improvement in diagnostic performance has not been backed up by a theoretical argumentation so far. In this report we show that better accuracy of Aβ42/40 compared to Aβ1-42 is granted by fundamental laws of probability. In particular, it can be shown that the dispersion of a distribution of a quotient of two random variables (Aβ42/40) is smaller than the dispersion of the random variable in the numerator (Aβ42), provided that the two variables are proportional. Further, this concept predicts and explains presence of outlying observations, i.e., AD patients with falsely negatively high Aβ42/40 ratio, and non-AD subjects with extremely low, falsely positive, Aβ42/40 ratio.

## 1. Introduction

Alzheimer’s disease (AD) is a complex neurodegenerative disorder characterized by progressive cognitive impairment such that activities of daily living are impacted, including alterations in spatial and temporal orientation and episodic memory loss. AD is the most common cause of cognitive decline in subjects over 65 years of age [1]. It is a growing global public health problem leading to serious concerns with severe implications for society. The prevalence usually doubles every five years after the age of 65 [2,3]. Currently about 6.2 million people in the USA are afflicted by AD, a number that is expected to grow to 13.8 million by 2060 [3]. It has been estimated that about 44 million people live with dementia worldwide and that this number may triple by 2050 due to the population ageing [4].

Pathologic alterations of AD begin in medial temporal lobe and the areas of neocortex decades before the onset of the clinical symptoms [5,6]. From a clinical perspective, AD progresses throughout three stages of (i) pre-symptomatic stage, (ii) prodromal stage, such as mild cognitive impairment (MCI), and eventually (iii) a symptomatic stage with dementia [7]. Approximately 10–20% of MCI patients convert to AD every year [8]. Since the clinical symptoms of the disease are usual preceded by preclinical phase (mainly symptom-free), early diagnosis of AD remains extremely difficult. AD biomarkers are usually tested when patient has already progressed to the MCI or even later stage. Therefore, studies have been undertaken to prove that assessment of the biomarkers in the Cerebrospinal Fluid (CSF) reasonably early predict progression of MCI to the dementia phase with accuracy of above 80% [9,10].

Pathophysiology of AD relies on the accumulation of amyloid beta (Aβ) plaques and neurofibrillary tangles, neuroinflammation, and glial activation. Extracellular senile plaques, consisting of Aβ peptides, and intracellular neurofibrillary tangles, composed of hyperphosphorylated form of Tau (pTau) molecules, have been shown to be the core neuropathological features in the central nervous system of AD patients [5]. Thus, these two groups of molecules are considered the best-validated AD biomarkers.

Aβ peptides, which are the main component of senile plaques, arise via enzymatic cleavage of β-amyloid precursor protein (APP) [11]. Aβ’s are formed by the sequential processing of APP via β-site amyloid precursor protein cleaving enzyme 1 (BACE1) and γ-secretase. Several isoforms of Aβ peptides are released [12]. The isoform of Aβ peptide ending at the amino acid position 42 (Aβ42) accounts for approximately 5–10% of the total Aβ isoforms in the CSF [13]. Of note, since N-terminus specific assays were not available in the past, in this paper we use Aβ1-42 when we talk about N- and C-termini specific assays, as they are mostly used today and reserving Aβ42 as an abbreviation for a generic term of assays not necessarily N-terminus specific. The mechanisms leading to the decreased concentrations of Aβ42 in the CSF of AD patients are still not fully understood. Some authors have suggested that reduced CSF concentrations might result from Aβ42 sequestration in AD plaques. Indeed, Aβ42 is a major component of the plaques in the brains of AD patients [14], and studies have indicated that the CSF Aβ42 correlates inversely with plaque load as found in autopsies or with positron emission tomography (PET) [15,16]. On the other hand, reduced CSF Aβ42 concentrations are also observed in other diseases, with plaques absent, such as bacterial meningitis [17]. Thus, presented theory does not explain fully a selective lowering in the CSF Aβ42 concentration. Possible hypotheses include reduction in the rate of Aβ42 generation, increased degeneration of Aβ42, or oligomerization of Aβ monomers [18,19].

Twenty years ago, the Lab for Clinical Neurochemistry in Erlangen was the first center worldwide that established the CSF Aβ42/40 concentration quotient, in addition to Aβ1-42 concentration, as a routine diagnostic biomarker in AD [20]. The inspiring idea came from previous work of one of us [21], which, to our best knowledge, inspired in turn Shoji et al. [22]. Despite the lack of promise for CSF Aβ40 as a biomarker per se, it became obvious almost immediately that the normalization of Aβ42 for the total amount of Aβ (represented by the most abundant isoform, Aβ40) is superior to that of Aβ42 alone. Consequently, around 2015 several centers worldwide started using Aβ42/40 as a routine diagnostic tool [23]. Since then, dozens of studies, as recently reviewed in [24], have reconfirmed improved performance of the Aβ42/40 ratio, compared to Aβ1-42, as a diagnostic and prognostic biomarker in AD. All these reports may be broadly categorized into three groups: (i) diagnostic studies for AD, including those using clinical diagnosis as reference (case control design), including those aiming at comparison to other modalities, such as amyloid PET as a proxy of AD pathology; (ii) studies on differential diagnosis across neurodegenerative disorders, focusing on differential diagnoses against AD; and (iii) prognostic studies, where the Aβ42/40 ratio was tested to predict progression from pre-clinical to dementia stage of the disorder. For example, concordance between Aβ PET and CSF biomarker concentration was observed with different Aβ PET tracers. An inverse, non-linear association between Aβ42, but not Aβ40, and amyloid PET using the Pittsburgh compound B (PiB) was shown in studies of both AD patients and cognitively normal individuals [15,25]. While high concordance between CSF Aβ42 levels and amyloid-β PET imaging is now well-established [26,27], discordance between CSF Aβ42 levels and PET imaging-positive results is also known. Such discordant results are more frequently observed in cognitively normal individuals [28,29], which leads to a speculation that the two modalities provide partially independent information [29]. The concordance of the CSF results with PET imaging highly significantly improves (from about 75% to about 90%) when Aβ42/40 ratio replaces Aβ42 as a reference [16,28,30]. Furthermore, evidence that CSF Aβ42 concentration decreases before amyloid-β is detectable with PET imaging suggests that the CSF Aβ42 is a more sensitive marker of AD at very early stages, while Aβ PET may be used for better grading of early AD [26].

CSF biomarkers, such as Aβ peptides, have been found to help in differentiation between AD and other types of neurologic conditions, such as non-AD dementia, which may have similar clinical symptoms [31,32]. For example, Aβ40, an isomer of not much utility in early AD diagnosis, was found decreased in Cerebral Amyloid Angiopathy (CAA) [33], FTD [34], vascular dementia (VaD), and Dementia with Lewy bodies (DLB) [30,35], compared to AD. Summarizing, Spies et al. reported both sensitivity and specificity metrics of larger than 80% when Aβ42/40 was used to differentiate AD from FTD, DLB, VaD, and other non-AD dementia diseases [35]. Hence, it becomes clear that Aβ42/40 is useful in differential diagnosis, provided the diagnosis question is properly formulated.

In light of all convincing empirical evidence briefly reviewed above, it is interesting that a very fundamental question has remained unanswered for almost two decades, namely, why is Aβ42/40 a better biomarker than Aβ1-42. This becomes particularly intriguing when we realize that it is only the numerator of the quotient in question (concentration of Aβ1-42) that is altered (decreased) in AD; the denominator (concentration of Aβ1-40) remains unaltered or, as sometimes observed, slightly and irrelevantly increased [36]. Hence, the question is, why normalizing of the Aβ1-42 concentration leads to a better separation of AD and non-AD subjects.

A biomarker *A* is considered as a superior biomarker than *B* when it characterizes with improved sensitivity and/or specificity, without disproving the other characteristic, or both. Several statistical measures are established to make this evaluation possible, including Youden index, area under the Receiver Operating Characteristic (ROC) curve, overall accuracy, and comparison of sensitivity at a fixed level of specificity or specificity at a fixed level of sensitivity. All those metrics are functions of the distributions of the biomarkers in question: the less overlap of the distributions of a given biomarker in two medical conditions, the better separation of the groups. Hence, to better understand that the improved diagnostic performance of Aβ42/40, compared to Aβ1-42, does not result from pure chance but rather is intrinsically linked to the fundamental laws of nature, we need to consider the distributions of the concentrations of the biomarkers and their ratio from the perspective of probability and mathematical statistics.

## 2. Theoretical Properties of the Variables

It is crucial to understand that for the following discussion no assumptions are made on the shape of the distributions of the underlying variables (like normal, skewed, etc.). Hence, the following derivation is equally valid for all distributions, theoretically considered or empirically observed.

**Figure 1:**
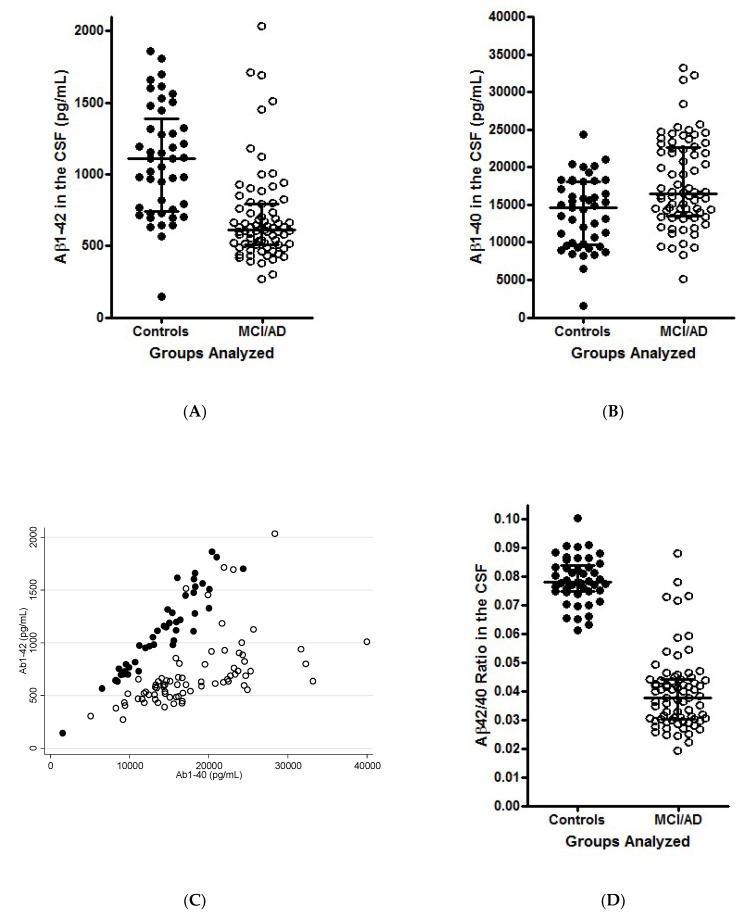
Scatterplot of the concentrations of Aβ1-42 (**A**) and Aβ1-40 (**B**) by groups (AD, open circles; Controls, closed circles). (**C**)correlation between the two biomarkers in AD (open circles, Spearman ρ = 0.73) and Control subjects (closed circles, Spearman ρ = 0.95). Scatter of the Aβ42/40 ratio in the two groups (**D**). In spite of highly significant decrease of Aβ1-42 in AD, a substantial overlap of the data is observed, which is much smaller in case of Aβ42/40. (**A**,**B**,**D**) reprinted, with modifications, from [36], (copyright IOS Press and the authors (2015)), with kind permission from IOS Press. The publication is available at IOS Press through http://dx.doi.org/10.3233/JAD-140771. (**C**) presents unpublished data from the same study.

First, we notice that the improved diagnostic performance of the Aβ42/40 ratio, compared to the Aβ1-42 concentration alone, can be better understood if we observe that the dispersion [variance (Var)] of the Aβ42/40 ratio is smaller than that of Aβ1-42 and Aβ1-40 (Figure 1A,B,D), and that Aβ1-42 and Aβ1-40 are proportional, conditional on the (patho)physiological status, i.e., presence or absence of AD (Figure 1C).

Indeed, from Equation (5) in Koop [37], we know that for random variables Z and U, with Z directly proportional to U

Var(Z) > [E(U)]^2^ Var(Z/U),

where variance (Var) is a measure of dispersion of a distribution, and E is the expectation of the random variable, i.e., its mean. Koop states that this inequality *“asserts that the variance of the linear estimator Z is greater than that of the ratio estimator E(U)(Z/U)”* [37]. Now, for E(U) > 1 we have:

Var(Z) > Var(Z/U).

This proves that the variance of the ratio of two directly proportional random variables (where the expectation of the denominator is at least 1) is smaller than that of the numerator. Translated to our context, this means that the variance (dispersion) of Aβ42/40 is less than the variance of Aβ1-42 given that Aβ1-42 and Aβ1-40 are proportional and that the mean of Aβ1-40 is greater than one, or

Var(Aβ1-42) > Var(Aβ42/40).

This means that the distribution of the ratio is more “compact” than the distribution of the random variable in the numerator (Aβ1-42).

## 3. Illustration of the Theoretical Findings

Let us illustrate the theoretical findings above on some simulated data. It needs to be stressed that this simulation, and the resulting figure, is presented exclusively for illustrative purpose, and should not be treated as a proof, which is derived above. To illustrate the line of the above argumentation, we generated ten thousand observations of (Aβ1-42, Aβ1-40) from a bivariate normal distribution for control and AD group (performed in *R*, version 3.6.1). Again, note that normality of the distribution is not a necessary assumption for the above argument to be valid. In the control group the mean (i.e., the expectation) of Aβ1-42 is 1200 and in the AD group it is 400, while the variance of Aβ1-42 is 160,000 in both groups (which is equivalent to standard deviation of 400). The mean of Aβ1-40 is 12,000 and its variance 16,000,000 in both groups (which corresponds to standard deviation of 4000), while the correlation between Aβ1-40 and Aβ1-42 is 0.9. Hence we have

$$\left(\begin{array}{c} A\beta 1-40\\ A\beta 1-42 \end{array}\right)\sim N\left(\left(\begin{array}{c} 12000\\ 1200 \end{array}\right),\;\;\left(\begin{array}{cc} 16,000,000 & 0.9\\ 0.9 & 160,000 \end{array}\right)\right)$$

for the control group, and

$$\left(\begin{array}{c} A\beta 1-40\\ A\beta 1-42 \end{array}\right)\sim N\left(\left(\begin{array}{c} 12000\\ 400 \end{array}\right),\;\;\left(\begin{array}{cc} 16,000,000 & 0.9\\ 0.9 & 160,000 \end{array}\right)\right)$$

for the AD group.

**Figure 2:**
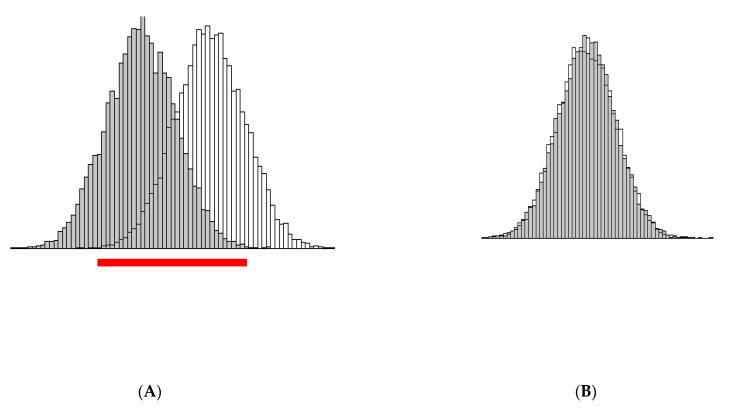

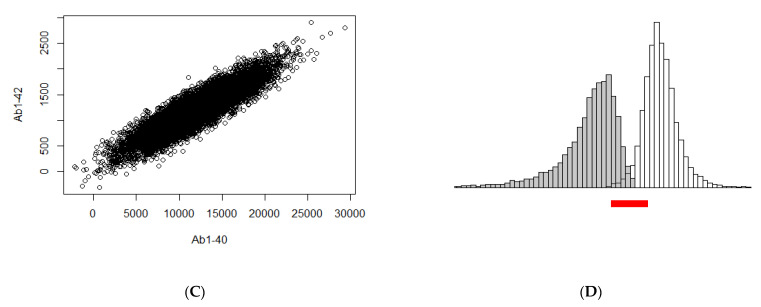
Illustration of the theoretical derivation with simulated data: (**A**) Histogram of the distribution of Aβ1-42 in AD (gray) and Controls (white) with obvious overlap marked by a red bar; (**B**). Histogram of the (overlapping) distribution of Aβ1-40 in AD and Controls; (**C**) Scatterplot of Aβ1-42 and Aβ1-40; (**D**) Histogram of the quotient of the two variables (i.e., Aβ42/40) in AD (gray) and Controls (white); obviously much smaller overlap of the two distributions, compared to that on Figure 2A, is seen (red bar). Further, both distributions are clearly denser around their respective expectations, due to the smaller dispersion.

A histogram of Aβ1-42 for both groups separately is provided in Figure 2A. From that figure it is clear that there is a shift in the distribution of Aβ1-42 and that there is some major overlap between the distributions (indicated by a red bar). Figure 2B presents the histogram of Aβ1-40, which overlay as both groups have a N(12000, 1600) distribution. Figure 2C provides a scatterplot of Aβ1-42 and Aβ1-40, from which we can clearly see the positive correlation (i.e., they are proportional). Finally, in Figure 2D we present a histogram of the ratio Aβ42/40 for both groups separately. From the Figure 2A, it becomes immediately clear that the variance of the Aβ42/40 ratio is much smaller than that of Aβ1-42 in both groups, and that the separation between the control and AD group is more pronounced (less overlap). Therefore, separation between control and AD group is easier on the Aβ42/40 scale than on the Aβ1-42 scale.

In general, if the distribution of a variable *X* (in our case, CSF concentrations of Aβ1-42) for the AD group is a shifted version (to the left, hence a negative shift) of the control group and the shift is denoted by *s >* 0 we can write $X_{AD}=X_{control}-s$. If further *Y* (in our case, CSF concentration of Aβ1-40) is a positive random variable, with the same distribution for the control and AD group (so $Y_{control}=Y_{AD}=Y)$, then

$$E\left(\frac{X_{AD}}{Y}\right)=E\left(\frac{X_{control}}{Y}\right)-s\;E\left(\frac{1}{Y}\right).$$

Note that if *Y* is a positive random variable, then *E(1/Y) > 0* and

$$E\left(\frac{X_{\;AD}}{Y}\right)<E\left(\frac{X_{control}}{Y}\right).$$

This clarifies the shift that is denoted on Figure 2D.

Further, if $X_{AD}$ and $X_{control}$ are proportional to *Y*

$$Var\left(\frac{X_{AD}}{Y}\right)<Var\left(X_{AD}\right)$$

and

$$Var\left(\frac{X_{control}}{Y}\right)<Var\left(X_{control}\right).$$

This is illustrated by the difference in variance (i.e., spread) of the distribution of the biomarkers in Figure 2A,D.

## 4. Conclusions

In spite of purely theoretical derivation, our argumentation provides a practical aspect for the diagnostically oriented interpretation of the biomarker data. A strong positive correlation of two variables implies that a large (small) value of one variable is linked to a large (small) value of the other variable in the majority of, but not necessarily in all, cases. In a sufficiently large study, subjects may be observed by pure chance with large value of one variable and average or perhaps even small values of the other variable, irrespective of their (patho)physiological status. This leads to extremely large or extremely small ratios of the two variables. Correspondingly, empirical observation of outliers, i.e., AD patients with falsely high Aβ42/40 ratio, or non-AD subjects with falsely low Aβ42/40 ratio, is a direct consequence of the distributions of the biomarkers discussed above. Taken together, on a probabilistic level, and irrespectively of otherwise important considerations of biochemistry and pathophysiology, we were able to prove that the ratio of two biomarkers that are proportional to each other separates two groups of subjects with different medical conditions better than a single of those biomarkers does. A possible explanation from the pathophysiological point of view might be that Aβ42/40 compensates for abnormally high or abnormally low total Aβ load in the CSF, therefore normalizing inter-individual variability of the CSF Aβ42 levels. The improved diagnostic performance of Aβ42/40 compared to Aβ1-42 in Alzheimer’s Disease is therefore granted by the fundamental laws of probability.
